# C-banding and AgNOR-staining were still effective complementary methods to indentify chromosomal heteromorphisms and some structural abnormalities in prenatal diagnosis

**DOI:** 10.1186/s13039-019-0453-1

**Published:** 2019-09-18

**Authors:** Jian Jiang Zhu, Hong Qi, Li Rong Cai, Xiao Hui Wen, Wen Zeng, Guo Dong Tang, Yao Luo, Ran Meng, Xue Qun Mao, Shao Qin Zhang

**Affiliations:** Prenatal Diagnosis Center, Beijing Haidian Maternal and Child Health Hospital, Beijing, 100080 People’s Republic of China

**Keywords:** Prenatal diagnosis, C-banding, AgNOR-staining, Chromosomal heteromorphisms, Chromosomal structural abnormality, Recurrent spontaneous abortion

## Abstract

**Background:**

In prenatal diagnosis, CMA has begun to emerge as a favorable alternative to karyotype analysis, but it could not identify balanced translocations, triploidies, inversion and heteromorphisms. Therefore, conventional cytogenetic and specific staining methods still play an important role in the work-up of chromosome anomaly. This study investigated the application of C-banding and AgNOR-staining techniques in prenatal diagnosis of chromosomal heteromorphisms and some structure abnormalities.

**Results:**

Among the 2970 samples, the incidence of chromosomal heteromorphisms was 8.79% (261/2970). The most frequent was found to be chromosome Y (2.93%, 87/2970), followed by chromosome 1 (1.65 %, 49/2970), 9 (1.52 %, 45/2970), 22 (0.77 %, 23/2970) and 15 (0.64 %, 19/2970). We compared the incidence of chromosomal heteromorphisms between recurrent spontaneous abortion (RSA) group and control group. The frequency of autosomal hetermorphisms in RSA group was 7.63% higher than that in control group (5.78%), while the frequency of Y chromosomal heteromorphisms was 4.76% lower than that in control group (5.71%). Here we summarized 4 representative cases, inv (1) (p12q24), psu dic (4;17) (p16.3;p13.3), r(X)(p11; q21) and an isodicentric bisatellited chromosome to illustrate the application of C-banding or AgNOR-staining, CMA or NGS was performed to detect CNVs if necessary.

**Conclusions:**

This study indicated that C-banding and AgNOR-staining were still effective complementary methods to identify chromosomal heteromorphisms and marker chromosomes or some structural rearrangements involving the centromere or acrocentric chromosomes. Our results suggested that there was no evidence for an association between chromosomal heteromorphisms and infertility or recurrent spontaneous abortions. Undoubtedly, sometimes we needed to combine the results of CMA or CNV-seq to comprehensively reflect the structure and aberration of chromosome segments. Thus, accurate karyotype reports and genetic counseling could be provided.

## Introduction

Currently, chromosomal microarray analysis (CMA) and copy number variation sequencing (CNV-Seq) are widely used in prenatal diagnosis due to the capability of identifying microdeletion and microduplication syndromes as well as *de novo* pathogenic CNVs which may be missed by conventional karyotyping [1]. However, their drawbacks are also obvious including the incompetence of balanced translocations, triploidies, inversion and heteromorphisms identification. So far, most of the clinical experts do not support the replacement of conventional karyotyping by CMA as a first-tier clinical test or in pregnancies at low risk for chromosome anomalies [2, 3] mainly due to the detection of variant of unknown significance (VOUS) and economic reasons. Therefore, the karyotype analysis technology is still essential for chromosomal disorder diagnosis.

After different treatments such as denaturation and/or enzymatic digestion, chromosomes show light and dark bands under light microscopy in different regions. Chromosome banding techniques are generally divided into two types: (1) bands are distributed along the entire chromosome, e.g. the most frequently used Giemsa-trypsin banding (G-banding), Q banding and R banding; (2) bands are located in specific chromosomal regions, including C-banding (constitutive heterochromatin), Ag-NOR staining (nucleolar organizer region) and T-banding (telomere). Each chromosome has a unique band pattern that can be reliably identified according to the corresponding banding technique. However, a dark-stained band by a particular treatment may appear light-stained in another. G-banding is the most frequently used method in the clinical laboratories because of its stability and cost-effectiveness.

C-banding and AgNOR-staining as the main banding techniques for chromosomal heteromorphisms detection are not conventional used in many laboratories due to the chromosomal heteromorphisms are generally considered to have no pathological significance and could be stably inherited to the offspring. Surprisingly, there have been increased conflicting evidence as to the possible association between heteromorphisms and the recurrent abortions [4–6], infertility or reproductive outcomes following assisted reproductive technology [7–9]. This study summarized the application of C-banding and Ag-NOR staining techniques for the prenatal diagnosis of fetal chromosome karyotype in our hospital, and explored the relationship between heteromorphisms and recurrent spontaneous abortion (RSA).

## Materials and methods

### Sample information

From May 2015 to April 2018, a total of 2972 cases accepted karyotype analysis by invasive prenatal diagnosis based on the indications of high-risk including advanced maternal age, positive aneuploidy screening results, or ultrasound structural anomalies in the Beijing Haidian Maternal and Child Health Hospital. The pregnant women’s obstetrical history was detail recorded, 2970 successful diagnostic samples were divided into two groups: the RSA group (history of spontaneous abortions≥2, Group A) and the control group (history of spontaneous abortions≤1, Group B). Samples with chromosomal heteromorphisms, structural abnormalities involving the centromere regions or acrocentric chromosomes, as well as mark chromosomes were verified by C-banding and AgNOR-staining techniques at first. Then CMA or NGS was also used to exclude chromosome CNVs if necessary. All clinical tests were based on written informed consent of pregnant women.

### Cell culture and karyotype analysis

Fetal cells obtained from villus, amniotic fluid or cord blood were cultured with double-line using standard methodologies. Chromosomes in metaphases were prepared from the cultured cell or lymphocytes using Giemsa-trypsin banding techniques. At least twenty metaphases chromosomes were counted and three metaphases chromosomes were analyzed in each line by two independent laboratory technicians to avoid uncertainty and variable results.

Chromosomal heteromorphisms were described following the criteria in the International System for Chromosome Nomenclature [10]. 1/9/16qh+ is defined as being at least twice the length of the corresponding region on the homologous chromosome, qh- is defined as being half length of the normal corresponding region. Polymorphisms of the Y chromosome were evaluated as such that Yqh+ (>size of chromosome 18), and Yqh- (<size of chromosome 21). For D/G –group chromosomes, the increase/decrease of the centromere or short arm in length were designated as cenh+/- if the variants were also C-banding positive. Additionally, distinct variants in size or number of satellites (ps+, pss) and lengths of stalks (pstk+) of the D/G –group were also recorded. The pericentric inversion of chromosomes 9 and Y were also categorized as heteromorphisms [8].

### C-banding

Conventional slide with metaphases chromosomes was incubated in 5% (saturated) barium hydroxide for about 1min ~ 5min (alkali treatment time varies with temperature and slide age) in 60°C water bath. After washing with tap water, the slide was subsequently incubated in two times the standard saline concentration (2×SSC) at 60°C for 90 min and it was finally stained for 15 minutes by the Giemsa dye.

### Ag-NOR staining

The slide was covered with 4 layers of clean lens paper (the size was slightly smaller than the slide) first. Then, it was moved to a plate floating in a 65°C water bath. 5ml of freshly prepared 10% AgNO_3_ solution (containing 0.1% formic acid) was dropped onto the lens paper several times by a straw and the slide remained in the water bath for about 15min until the lens paper turned black or dark brown. After gently removing the lens paper with tweezers, the slide was washed with tap water and samples were finally stained with Giemsa for 1 minute in room temperature. The most important thing in the whole process of the experiment was to avoid the light.

### CMA and CNV-seq

Affymetrix CytoScan 750K array platforms were used to detect copy number variants. Genomic DNA extraction was performed using Genomic DNA Extraction kit (QIAamp DNA Blood Mini Kit, QIAGEN GmBH, Hilden, Germany) according to the in-house protocols. The standard experimental procedure incorporated the following steps: digestion, ligation, polymerase chain reaction (PCR), PCR purification, fragmentation, labeling, hybridization, washing, staining and scanning. Data was analyzed using the Chromosome analysis software (Chromosome Analysis Suite version 2.1) (Affymetrix; Thermo Fisher Scientific, Inc.). Copy number variation sequencing (CNV-seq) was performed as previously described [11, 12].

The conventional genomic and phenotype public databases such as UCSC Genome Browser (http://genome.ucsc.edu/cgi-bin/hgGateway), ClinGene (http://www.clinicalgenome.org/), OMIM (http://omim.org), DECIPHER (https://decipher.sanger.ac.uk), DGV (http://dgv.tcag.ca/dgv/app/home) and PubMed (http://www.ncbi.nlm.nih.gov/pubmed) were used for retrieval and interpretation.

### Statistical analysis

Data were analyzed using SPSS Statistics (version 22.0). Differences between the RSA and the control groups within the cohort were tested with Chi-squared test statistics. The significance level was set at *p* < 0.05.

## Results

Among the 2972 prenatal diagnosis cases (including 2735 amniotic fluid, 68 villus and 169 cord blood), 2 cases of amniotic fluid failed to culture due to less cloning, and the success rate of cultivation was 99.93% (the proportion of male and female was 1533:1437). A total of 131women were investigated in the group A, and the group B consisted of 2839.

### Chromosome heteromorphisms

**Fig. 1:**
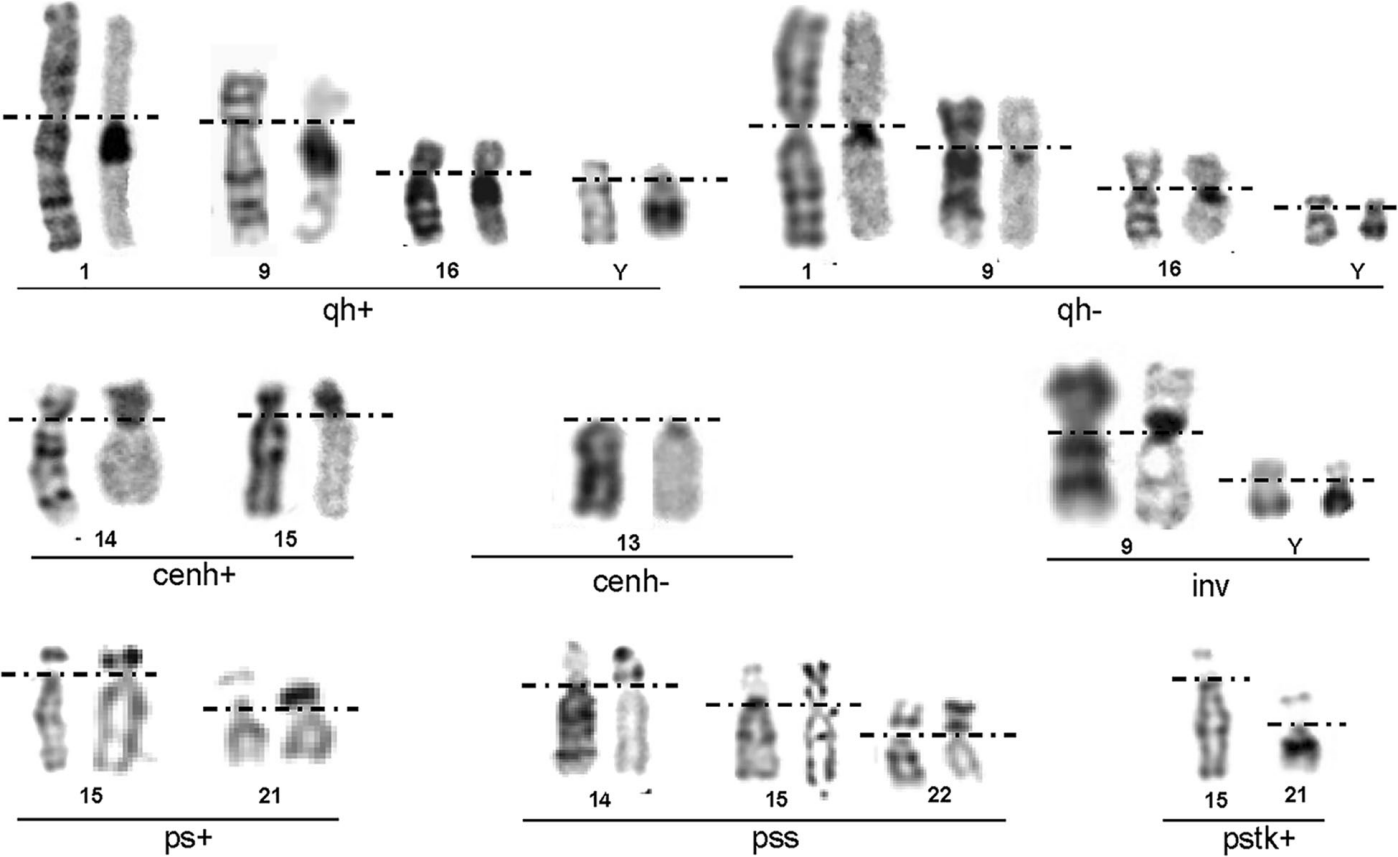
Partial karyotypes of chromosomal heteromorphisms by G-banding and C/AgNOR-staining

The distribution among different chromosomes heteromorphisms was presented in Table 1 with total frequency of 8.79% (261/2970). The most frequent was found to be chromosome Y (2.93%, 87/2970), followed by chromosome 1 (1.65 %, 49/2970), 9(1.52 %, 45/2970), 22 (0.77 %, 23/2970) and chromosome 15 (0.64 %, 19/2970). In 1533 male fetuses, 5.68 % (87/1533) had Y chromosomal heteromorphisms, followed by Yqh- 3.26 %, Yqh+ 2.09 %, and inv(Y) 0.39% (Table 3). The partial karyotypes of chromosomal heteromorphisms by G-banding and C/AgNOR-staining are presented in Fig. 1. Parental studies had been performed for most prenatally reported chromosome heteromorphisms, particularly for pericentric inversion, confirming that these variations were stably inherited in family.

**Table 1:** Summary the number and frequency of all observed Chromosomal heteromorphisms

Heteromorphisms types	Chromosome number										
	1	9	16	19	13	14	15	21	22	Y	Total (n; %)
qh+	44	15	10	1	-	-	-	-	-	32	102 (3.43%)
qh-	5	2	0	0	-	-	-	-	-	49	56 (1.89%)
ps+	-	-	-	-	1	-	7	9	14	-	31 (1.04%)
pss	-	-	-	-	-	1	-	1	1	-	3 (0.1%)
pstk+	-	-	-	-	3	5	1	4	5	-	18 (0.61%)
cenh+/-	-	-	-	-	0/1	1/0	11/0	0/1	3/0	-	17 (0.57%)
inv	-	28	-	-	-	-	-	-	-	6	34 (1.14%)
Total (n; %)	49 (1.65%)	45 (1.52%)	10 (0.34%)	1 (0.03%)	5 (0.17%)	7 (0.24%)	19 (0.64%)	15 (0.51%)	23 (0.77%)	87 (2.93%)	261 (8.79%)

The group A had a higher frequency (7.63%) of autosomal hetermorphisms compared with the group B (5.78%)(Table 2), but a lower frequency (4.76%) of Y chromosomal heteromorphisms compared with the group B (5.71%) (Table 3). While there were no statistically significant difference between two groups (*P* > 0.05).

**Table 2:** Number and frequency of autosomal heteromorphisms in different groups

Group	Chromosome heteromorphisms					
	No. of cases	1/9/16/19qh±	D/G variation	inv(9)	Total of heteromorphisms (n; %)	Chi-square test
Group A^a^	131	4 (3.05%)	4 (3.05%)	2 (1.53%)	10 (7.63%)	Pearson χ^2^=0.783 *p*>0.05
Group B^b^	2839	73 (2.57%)	65 (2.29%)	26 (0.92%)	164 (5.78%)	
Total	2970	77 (2.59%)	69 (2.32%)	28 (0.94%)	174 (5.86%)	

*Significant at *p* < 0.05.

^a^the recurrent spontaneous abortion group

^b^the control group

**Table 3:** Number and frequency of Y Chromosomal heteromorphisms in different groups

Group	Chromosome heteromorphisms					
	No. of male cases	Yqh+	Yqh-	inv (Y)	Total of heteromorphisms (n; %)	Chi-square test
Group A^a^	63	0	2 (3.17%)	1 (1.59%)	3 (4.76%)	Continuity Correction χ^2^=0.002 *p* > 0.05
Group B^b^	1470	32 (2.18%)	47 (3.2%)	5 (0.34%)	84 (5.71%)	
Total	1533	32 (2.09%)	49 (3.2%)	6 (0.39%)	87 (5.68%)	

*Significant at *p* < 0.05

^a^the recurrent spontaneous abortion group

^b^the control group

### Chromosome abnormal

C-banding and AgNOR-staining were important banding methods to characterize marker chromosomes or other structural rearrangements involving the centromere or acrocentric chromosomes. Here we summarized 4 representative cases, inv (1) (p12q24), psu dic (4;17)(p16.3;p13.3), r(X)(p11;q21) and an isodicentric bisatellited chromosome to illustrate the application of C-banding or AgNOR-staining. CMA or NGS was performed to detect CNVs. Among them , 2 CNVs with VOUS were detected in case#1, pathogenic CNVs were detected in case #2 and case #3, respectively (Table 4).

**Table 4:** C-banding or AgNOR-staining applied to the auxiliary diagnosis of 4 cases with chromosome abnormal

Case No.	Brief clinical information	Chromosome karyotype	SNP-array/CNV-seq	Pregnancy outcome
Case#1	42-year-old, G5P1,amniocentesis at 19 weeks’ gestation because of advanced age	**F:**46,XN,inv(1)(p12q24)mat **P:** 46,XX,inv(1)(p12q24) **H:**46,XY	F::arr [hg19] 2q13(110,498,141_110,980,295)x4, 12p12.1 (23,797,551_24,076,457)x1 P: arr [hg19] 2q13(110,498,141_110,980,295)x4 H: arr(1-22)x2,(XN)x1	continued pregnancy
Case#2	25-year-old, G5P1,amniocentesis at 18 weeks’ gestation because of a positive serological screening result (a high risk of Down syndrome 1:176)	**F:**45,XY,der(4)dup(4)(p15.1p16) psu dic(4;17)(p16.3;p13.3),-17 **P:**46,XX **H:**46,XY	F:arr [hg19] 2q12.3q13(107,586,661_110,980,295)x3, 4p16.3p15.1 (68,345_32,437,069)x3	induced abortion
Case#3	35-year-old, G4P1,puncture of umbilical vein at 26 weeks’ gestation because of advanced age and ultrasonic anomalies (coarctation of the aorta? long bone dysplasias).	**F:**45,X [31]/46,X,r(X)(p11;q21) [29] **P:**46,XX **H:**46,XY	F:seq[GRCh37]del(X)(p22.33p11.3),del(X)(q21.31q28) ChrX:g.60001_43660000del, 0710001_155260000del	induced abortion
Case#4	37-year-old, G2P1,amniocentesis at 19 weeks’ gestation because of advanced age	**F:**47,XN,+mar [37]/46,XN[63] **P:**46,XX **H:**46,XY	F:arr(1-22)x2,(XN)x1	continued pregnancy

Abbreviations: *F* Fetus, *P* Pregnant woman, *H* Husband of pregnant woman

## Discussion

Chromosomal variation were mainly refers to the variations on heterochromatic segments, satellites and satellite stalks in the population, but euchromatic heteromorphisms with C-banding and Ag-NOR negative could also be included [13–15]. Like most other literatures, here we mainly focused on the C-banding or AgNOR-staining positive chromosomal heteromorphisms.

As anticipated, while most of chromosmal heteromorphisms were successfully detected according to our criteria, we still occasionally encounter disputes between experienced technicians occasionally. Without a reference standard other than between homologues, it is difficult to determine whether two similar-sized homologues are slightly smaller or larger than the normal ones [16]. In addition, we found that the diagnostic criteria in different literatures were not uniform, although most of the literature was consistent with ours [8, 17], some did not mention the details of the judgment criteria [9, 18]. This could be one of the reasons for conflicting reports of heteromorphisms impact human reproduction. Shivanand [16] considered that the length of short arms of chromosome 16 was not appreciably altered by compaction, Moreover, it is intermediate in size compared to the qh regions of 1, 9,and 16. Also, it is easily identified in a C-banded cell, therefore provides a useful reference standard to set criteria. Frequencies of heteromorphisms in various populations showed differences due to ethnic origins, age and geographical distribution. Like other studies of Asian populations had shown greater variation in the size of the Y than in the white population [19]. In our study, the frequency of Y chromosome heteromorphisms was the highest, which was apparently higher than other literature [6] and Yqh-was slightly more common than Yqh+. Perhaps the populations selected introduced biases and the criteria used in current studies were subjective, making it difficult to directly compare frequencies. Our data showed that each frequency of chromosomal heteromorphisms does not appear to be significantly different in group A and group B, confirmed that variation in these C-banding or AgNOR-staining positive regions has no clinical significance. It was almost certain that the common heteromorphisms were stably inherited in the family by study of the parental chromosomes, which would strengthen the argument against the associated between heteromorphisms and infertility. However, further research is required to delineate the mechanisms impacting human reproduction, focusing on patients with unexplained infertility, poor embryonic development, and spontaneous abortions. Current cytogenetics has mainly focused its efforts on the identification of clonal chromosomal aberrations (CCAs). However, many investigators have demonstrated that “non clonal chromosome aberrations,” or NCCAs are not “noise” but rather a highly significant feature of the genome system, and the significance of NCCAs will emphasize the ultimate importance of studying heterogeneity in biology, including heteromorphisms and euchromatic variants [20]. Of equal importance, CNV-seq and CMA will complement the karyotyping method by allowing the detetion of small CNV, with more potentially pathogenic genetic candidates detected among the CNV regions, refined genetic causes of conditions like spontaneous miscarriage and other medical syndromes could be investigated [12, 21].

**Fig. 2:**
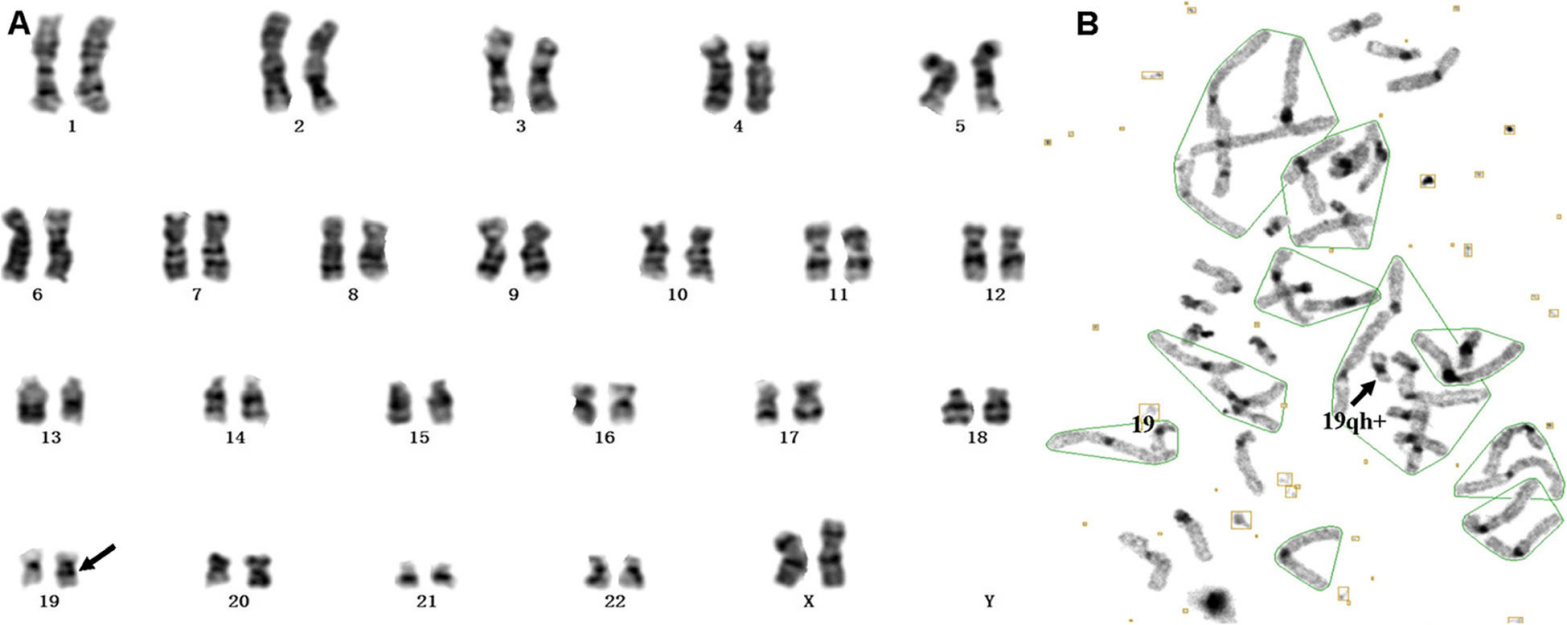
Prenatal diagnosis of chromosome 19 heteromorphism by G-banding (**a**) and C-banding (**b**). Chromosome 19 is labelled, with an arrow indicating the large heterochromatic region

In recent years, more and more studies had focused on the chromosomal heteromorphisms [22] which can be verified by the application of C-banding and AgNOR**-**staining techniques. Several other centromeric variants, verified by C-banding in the course of a prenatal diagnosis [23–28], have been described for chromosomes 5, 6, 12, 18, 19 or 20. Our data presented a rare heteromorphism on chromosome 19 and it was different from the four different classes that Crossen noted [29]. A distinct dark-staining band at the long arm of chromosome 19 near the centromere region was revealed by G-banding (Fig. 2a) and it was also dark-stained by C-banding as the same as centromere (Fig. 2b). So it was most likely constitutive heterochromatin which was considered without pathological significance. As the fetus has no abnormal ultrasound findings except for increased nuchal translucency (3.7mm) at 13 weeks’ gestation, the parents rejected further chromosome verification and CNVs testing. Our karyotype report was 46, XN, 19qh+. No abnormalities were found in the 6-month follow-up after birth. Indeed, the discovery of rare chromosomal polymorphisms raised the question of whether or not differences in size and banding pattern observed between homologue could account for the existence of a normal variant. Therefore, for rare chromosomal heteromorphisms, family verification and original analysis should be performed to better assess the genetic effects of the heteromorphisms. In addition, whether the heteromorphisms will change (such as changes in the length or size of heteromorphisms regions) during the genetic processes remains to be further studied.

**Fig. 3:**
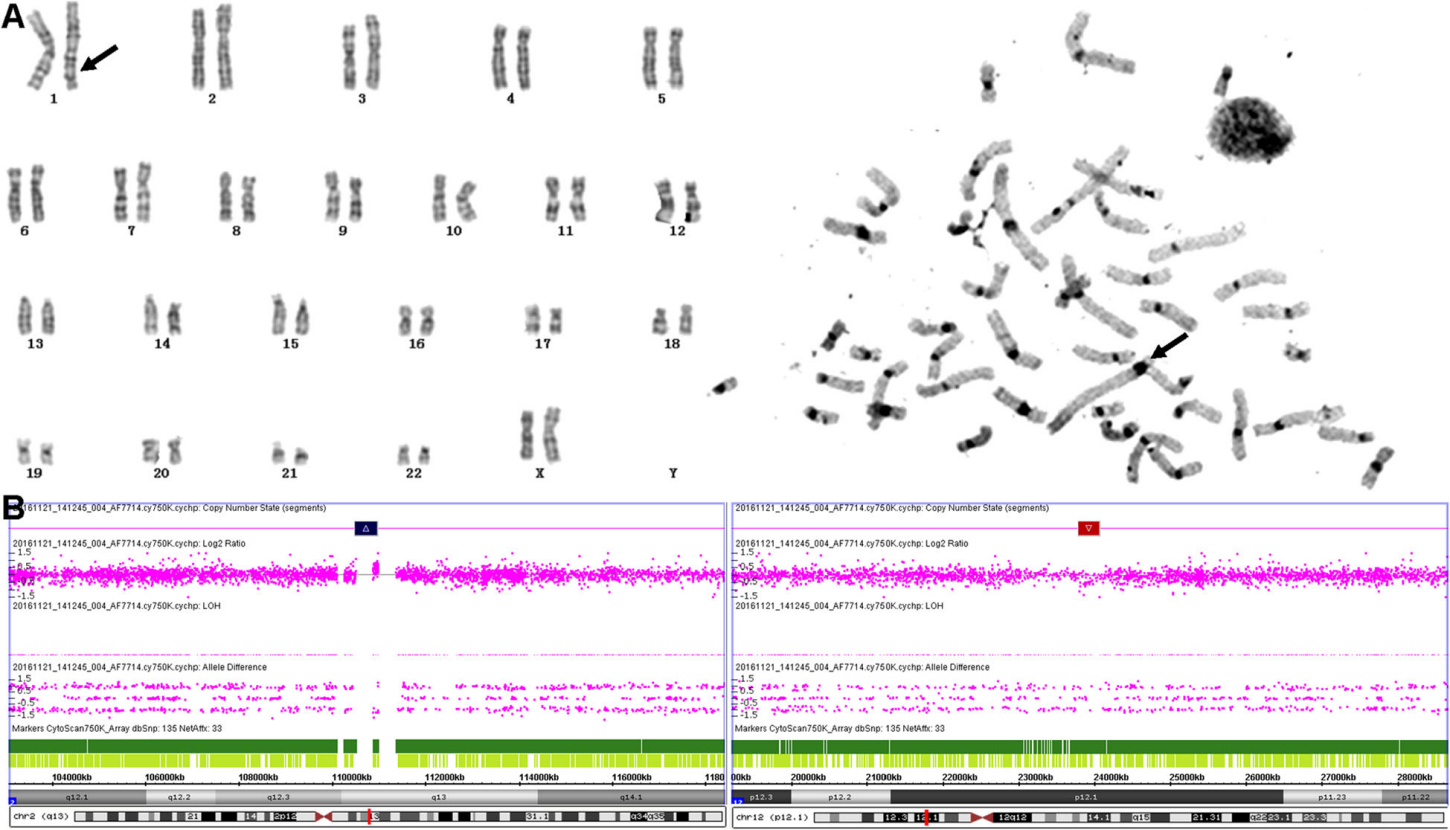
Prenatal diagnosis of 46,XX,inv (1)(p12q42) in Case #1. Chromosome 1 is labeled, with an arrow indicating the abnormal region. (**a**) G-banding (Left) and C-banding karyotypes (Right). (**b**) SNP-array analysis revealed 2q13 double duplication of and 12p12.1deletion of

In our prenatal diagnosis work, C-banding was performed to verify the pericentric inversion and structure rearrangement involving the centromere regions. It was convenient and easy to make diagnosis by observing the position and morphological changes of centromeres with C-banding. Our data showed that several cases of inv (9) cannot be clearly diagnosed by G-banding due to the short and poor karyotype, but it can be easily confirmed by C-banding (Fig. 1). Similarly, in the diagnosis of inv (1)(p12q42) (Case #1, Fig 3), the qh regions of inv (1) appeared in the distal long arm of the 1 chromosome rather than in the middle were especially visible by C-banding (Fig. 3a), two VOUS were revealed by Array-SNP (Fig. 3b). After genetic counseling, the pregnant woman chose to continue pregnancy with some misgivings. Fortunately, the child had no abnormalities since birth at term. We will continue to monitor the child's follow-up to check whether these VOUS really didn’t have clinical significance.

**Fig. 4:**
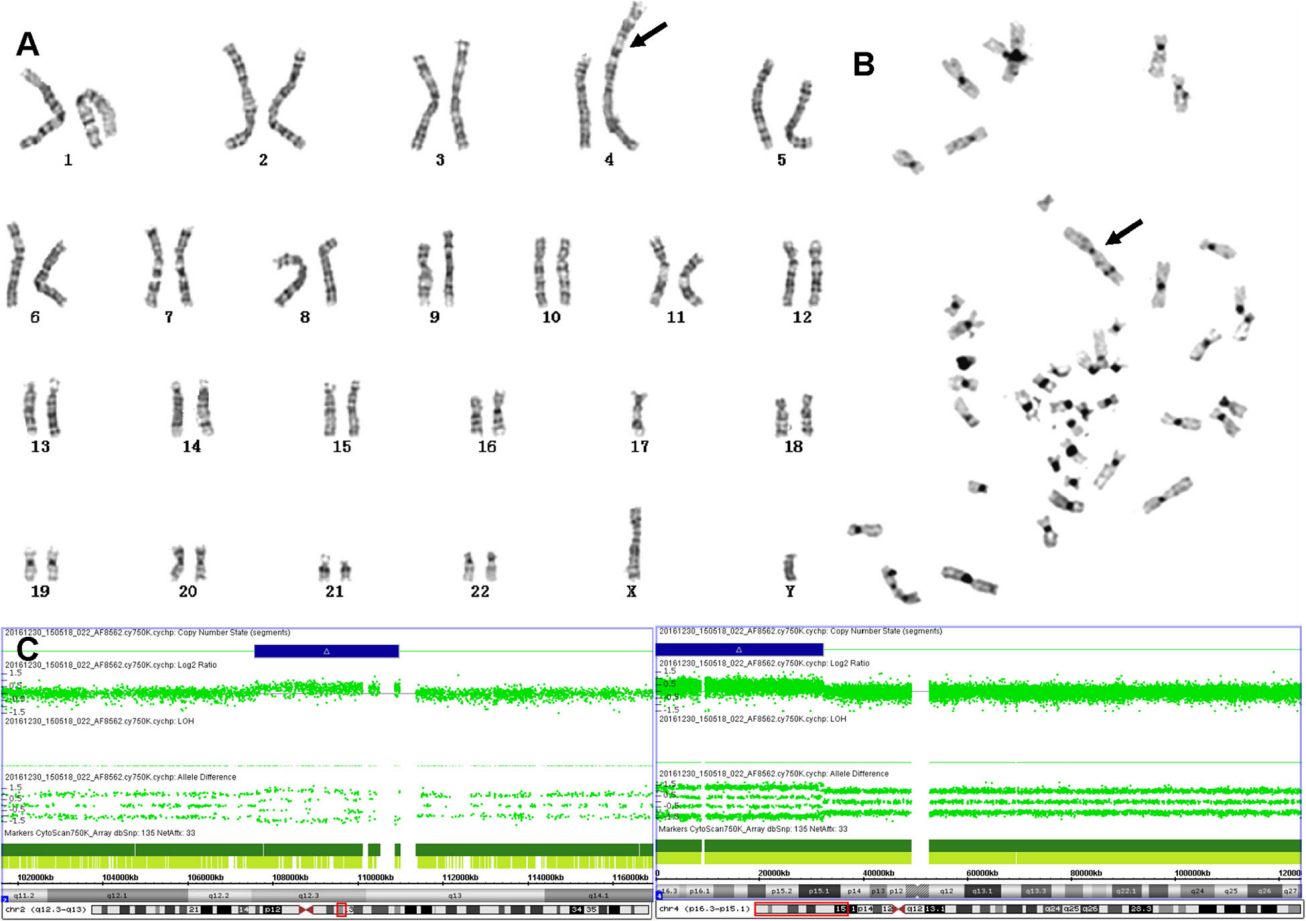
Prenatal diagnosis of 45,XY,der (4) dup (4)(p15.1p16) psu dic (4;17)(p16.3;p13.3),-17 in Case #2. Abnormal chromosome is indicated by arrows. (**a**) G-banding karyotype. (**b**) C-banding karyotype. (**c**) The SNP-array analysis revealed 2q12.3q13 and 4p16.3p15.1 duplications

As shown in the diagnosis of case #2, C-banding can also be used to identify pseudodicentric chromosome and to determine whether a fragment of unknown origin is heterochromatin. G-banding showed an apparent unknown dark-stained band (Fig. 4a, indicated by arrow) inserted at the breakpoint of the derived chromosome formed by reciprocal translocation of chromosomes 4 and 17. The C-banding demonstrated that the derivative chromosome was dicentric with only one primary constriction (presumably the active centromere), whereas the other regions were light stained (Fig. 4b). So we confirmed that the inserted fragment was not constitutive heterochromatin. Therefore, we conducted the CMA test and finally revealed that it was a 32.3 Mb duplication of 4p16.3p15.1, at the same time, a 3.3Mb duplication of 2q was also detected (Fig. 4c). The pregnancy was terminated at 23 weeks of gestation due to the chromosome abnormality.

**Fig. 5:**
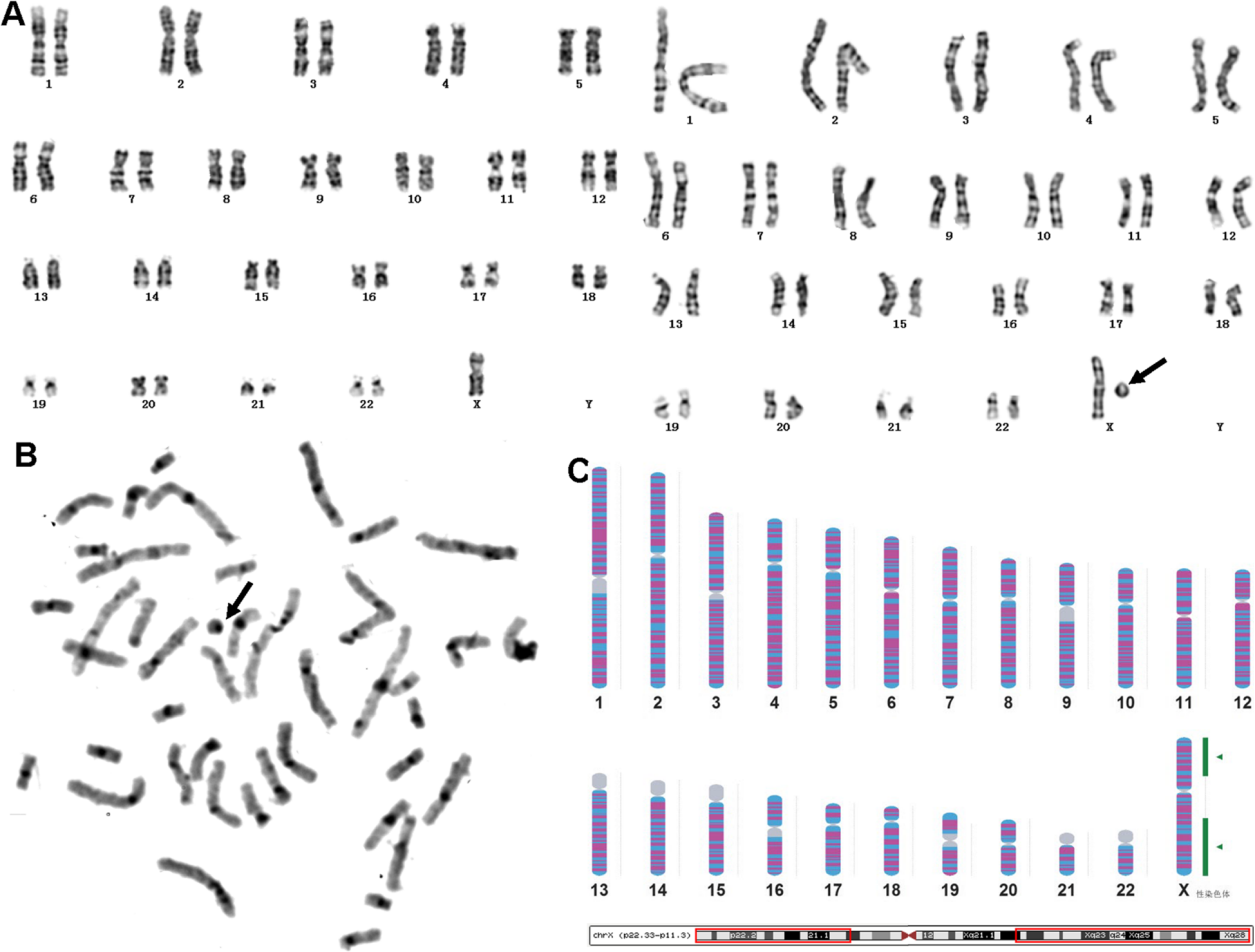
Prenatal diagnosis of 45,X [31]/46,X,r(X)(p11;q21) [29] in Case #3. Abnormal chromosome is indicated by arrows. (**a**) G-banding Karyotype. (**b**) C-banding Karyotype . (**c**) CNV-seq analysis revealed large fragment deletions in chromosome X deletion. The gray baseline beside the chromosome represent the copy number of chromosomes was normal, green portions of the baseline represented the regions in chromosome were deletions

**Fig. 6:**
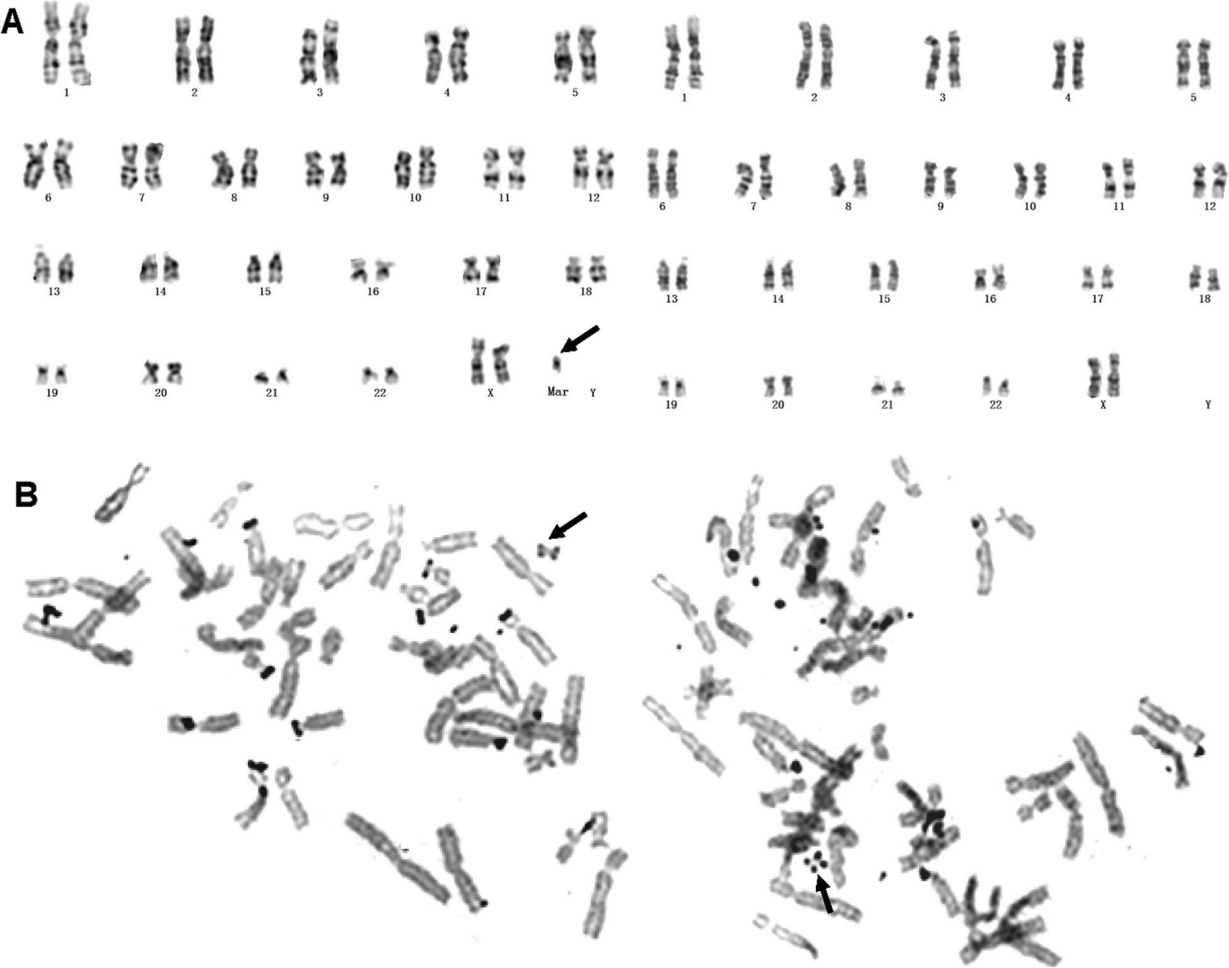
Prenatal diagnosis of 47,XN,+mar [37]/46,XN [63] in Case#4. There is no abnormal detected by SNP-array (Data not shown). The marker chromosome is indicated by arrows. (**a**) G-banding Karyotype. (**b**) AgNOR-staining Karyotype

C-banding and AgNOR**-**staining were also routinely used to characterize marker chromosomes. In case #3, Karyotyping revealed mosaic of the mark chromosome with two types of cell lines 45,X and 46,X,+mar (Fig. 5a). The C-banding showed that the mark chromosome had a clear dark-staining centromere and the rest of the chromosome regions were light stained (Fig. 5b). Therefore, we hypothesized that the mark could not be the heterochromatic region of the Y chromosome but likely to be a circle X with a large fragment deletion, and this was later confirmed by the CNV-seq analysis (Fig. 5c). About 16% of the individuals with Tuner syndrome have an addtional cell line with 46 chromosomes due to the presence of an extra ring chromosome X [30]. Ring X chromosome, with different degree of mosaicism, showing common clinical characteristics of Turner syndrome had been reported, and karyotype-phenotype correlation related manifestation was dependent on the degree of genetic material lost in ring (X) formation as well as mosaicism [31]. Undoubtedly, for mosaic Tuner with mixed cell lines, FISH tests for Xcen and Ycen should be performed since ring X is more commonly seen in mosaic Turner and present of Y material will be clinically significant and requires intervention. In addition, the interphase FISH of uncultured cells can avoid the influence of cell culture and thus more accurately identify the degree of mosaicism, the metaphase FISH can clearly locate the recombinant chromosome. CNV-seq or CMA analysis can reveal exact break points on both arms of X chromosomes. In contrast to case **#**3, the supernumerary marker chromosomes (SMC) of case #4, which we highly speculated that it was a bisatellited metacentric microchromosome (Fig. 6a). The further identified by AgNOR-banding showed prominent satellites on both sides of the marker (Fig. 6b). About 70% of SMC are derived from acrocentric chromosomes and those markers derived from can be identified by FISH [32].

CMA has advantages over conventional cytogenetic, including the ability to precisely characterize CNVs associated with abnormal karyotypes. Moreover, a significant proportion of cases studied by array detected clinically significant CNVs even in samples with apparently normal karyotypes [33, 34], but the expensive inspection costs and VOUS still require us to consider its cost-effectiveness. These scenarios present a challenge for prenatal diagnosis, and genetic counseling prior to prenatal CMA greatly facilitates delivery of complex results [35]. Although VOUS results have limited impact on parental well-being and perception of children’s development, the initial diminished perception of child competency and later dissatisfaction with genomic testing indicate the need to assist parents in coping with ambiguous results [36]. It is suggested to not change the current policy of microarray application in prenatal diagnosis until more data on the clinical significance of copy number changes are available [2]. So, without professional genetic counseling qualifications and informed consent of subjects, it is not appropriate to blindly expand the scope of detection without clinical indications. We recommend that CMA or CNV-seq used for fetal chromosomal structural abnormalities (especially *de novo*), ultrasound abnormalities, and the SMCs with C-banding and Ag-NOR negative. We also strongly recommended de novo SMC for CNVs examination (case #4) because that a prospective study showed that 69% of de novo SMC contained euchromatin material, 95.4% of which for non-acrocentric markers [37].

## Conclusion

Each technology has its limitations, we didn’t demonstrate that C-banding and/or AgNOR-staining was absolutely necessary above other available technologies such as FISH or CMA. But base on our work, it is suggested that C-banding and AgNOR-staining, which were more convenient, fast and economical, could still be effective complementary methods to study heteromorphic variations, and to characterize marker chromosomes or other structural rearrangements involving centromere regions or acrocentric chromosomes, especially when it was difficulties to make a definite diagnosis due to the short and/or poor G-banding karyotype. On the other hand, purely using G-banding without combining C-banding and AgNOR can hardly tell the difference between cenh+ and ps+, as well as pss and pstk+, and thereby causes the unreliable karyotype results.

Unsurprisingly, further studies are needed to delineate whether heteromorphisms impact human reproduction. Absolutely, there is an urgent need to establish normalized diagnostic criteria for chromosomal heteromorphisms, and this is essential if any degree of comparison is to be made between these and other clinical studies. Contrary to some previous studies, we found no evidence for an association between chromosomal heteromorphisms and infertility or RSA. Our study agreed that familial cytogenetic studies do not appear to be routinely necessary when a common chromosomal heteromorphisms is diagnosed prenatally [38]. Perhaps most critically, followup studies are distinctly lacking. Studies of the progeny (or products of conception) of carriers of heteromorphic variants are sorely needed to better delineate the heritability and consequences of these variants [22].

In summary, in prenatal diagnosis, it is necessary make a diagnosis in a limited time according to the limited clinical manifestations of the fetus, so reasonable detection methods should be selected and avoided the unnecessary expansion of detection scope which generated additional and more complex data to amplify and exacerbate some pre-existing ethical problems [39].

## Data Availability

Data supporting the results reported in the published article can be found in the tables and figures.

## Abbreviations

CCAs: Clonal Chromosomal Aberrations
CMA: Chromosomal Microarry Analysis
CNV-Seq: Copy Number Variation Sequencing
FISH: Fluorescence In Situ Hybridization
NCCAs: Non Clonal Chromosome Aberrations
NOR: Nucleolar Organizer Region
RSA: Recurrent Spontaneous Abortion
SMC: Supernumerary Marker Chromosomes
VOUS: Variant Of Unknown Significance

## References

[1] Wapner RJ, Martin CL, Levy B, Ballif BC, Eng CM, Zachary JM (2012). Chromosomal microarray versus karyotyping for prenatal diagnosis [J]. N Engl J Med.

[2] Miny P, Wenzel F, Tercanli S, Filges I (2013). Chromosomal Microarrays in Prenatal Diagnosis: Time for a Change of Policy?[J]. Microarrays (Basel).

[3] Novelli A, Grati FR, Ballarati L, Bernardini L, Bizzoco D, Camurri L (2012). Microarray application in prenatal diagnosis: a position statement from the cytogenetics working group of the Italian Society of Human Genetics (SIGU), November 2011[J]. Ultrasound Obstet Gynecol.

[4] Akbas H, Isi H, Oral D, Turkyilmaz A, Kalkanli-Tas S, Simsek S (2012). Chromosome heteromorphisms are more frequent in couples with recurrent abortions [J]. Genet Mol Res.

[5] Wang Y, Li G, Zuo MZ, Fang JH, Li HR, Quan DD (2017). Y chromosome polymorphisms may contribute to an increased risk of male-induced unexplained recurrent miscarriage [J]. Biosci Rep.

[6] Dai R, Pan Y, Fu Y, Liu Q, Han W, Liu R (2018). Role of male genetic factors in recurrent pregnancy loss in Northeast China [J]. Eur J Obstet Gynecol Reprod Biol.

[7] Cheng R, Ma Y, Nie Y, Qiao X, Yang Z, Zeng R (2017). Chromosomal polymorphisms are associated with female infertility and adverse reproductive outcomes after infertility treatment: a 7-year retrospective study [J]. Reprod Biomed Online.

[8] Hong Y, Zhou YW, Tao J, Wang SX, Zhao XM (2011). Do polymorphic variants of chromosomes affect the outcome of in vitro fertilization and embryo transfer treatment?[J]. Hum Reprod.

[9] Dong Y, Jiang YT, Du RC, Zhang HG, Li LL, Liu RZ (2013). Impact of chromosomal heteromorphisms on reproductive failure and analysis of 38 heteromorphic pedigrees in Northeast China [J]. J Assist Reprod Genet.

[10] Shaffer LG, Mc Gowan-Jordan J, Schmid M, International Standing Committee on Human Cytogenetic Nomenclature (2013). ISCN 2013 : an international system for human cytogenetic nomenclature (2013)[M].

[11] Liang D, Lv W, Wang H, Xu L, Liu J, Li H (2013). Non-invasive prenatal testing of fetal whole chromosome aneuploidy by massively parallel sequencing [J]. Prenat Diagn.

[12] Qi H, Xuan ZL, Du Y, Cai LR, Zhang H, Wen XH (2018). High resolution global chromosomal aberrations from spontaneous miscarriages revealed by low coverage whole genome sequencing [J]. Eur J Obstet Gynecol Reprod Biol.

[13] Jalal SM, Schneider NR, Kukolich MK, Wilson GN (1990). Euchromatic 16p+ heteromorphism: first report in North America [J]. Am J Med Genet.

[14] Webb GC, Krumins EJ, Eichenbaum SZ, Voullaire LE, Earle E, Choo KH (1989). Non C-banding variants in some normal families might be homogeneously staining regions [J]. Hum Genet.

[15] Song XH, Hsu HK, Su MT, Chang TS, Su PY, Chen M (2017). Euchromatic variants of 8q21.2 in twins [J]. Taiwan J Obstet Gynecol.

[16] Patil SR, Lubs HA (1977). Classification of qh regions in human chromosomes 1, 9, and 16 by C-banding [J]. Hum Genet.

[17] Sun L, Chen ZH, Yang L, Yi CX, Liu J, Ou CQ (2018). Chromosomal polymorphisms are independently associated with multinucleated embryo formation [J]. J Assist Reprod Genet.

[18] Inan C, Sayin NC, Dolgun ZN, Gurkan H, Erzincan SG, Uzun I, et al. Prenatal diagnosis of chromosomal polymorphisms: most commonly observed polymorphism on Chromosome 9 have associations with low PAPP-A values()[J]. J Matern Fetal Neonatal Med. 2017:1–8. 10.1080/14767058.2017.1416079 29262756

[19] Hou JW, Wang TR (1999). Study of human Y chromosome polymorphism in Taiwan [J]. Acta Paediatr Taiwan.

[20] Heng HH, Regan SM, Liu G, Ye CJ (2016). Why it is crucial to analyze non clonal chromosome aberrations or NCCAs?[J]. Mol Cytogenet.

[21] Bagheri H, Mercier E, Qiao Y, Stephenson MD, Rajcan-Separovic E (2015). Genomic characteristics of miscarriage copy number variants [J]. Mol Hum Reprod.

[22] Tempest HG, Simpson JL (2017). Why are we still talking about chromosomal heteromorphisms?[J]. Reprod Biomed Online.

[23] Fineman RM, Issa B, Weinblatt V (1989). Prenatal diagnosis of a large heteromorphic region in a chromosome 5: implications for genetic counseling [J]. Am J Med Genet.

[24] Jabs EW, Carpenter N (1988). Molecular cytogenetic evidence for amplification of chromosome-specific alphoid sequences at enlarged C-bands on chromosome 6[J]. Am J Hum Genet.

[25] Chantot-Bastaraud S, Siffroi JP, Berkane N, Heim N, Herve F, Uzan S (2003). Prenatal diagnosis of a large centromeric heteromorphism of chromosome 12: implications for genetic counseling [J]. Fetal Diagn Ther.

[26] Pittalis MC, Santarini L, Bovicelli L (1994). Prenatal diagnosis of a heterochromatic 18p+ heteromorphism [J]. Prenat Diagn.

[27] Friedrich U (1985). Centromere heteromorphism in chromosome 19[J]. Clin Genet.

[28] Petersen MB (1986). Rare chromosome 20 variants encountered during prenatal diagnosis [J]. Prenat Diagn.

[29] Crossen PE (1975). Variation in the centromeric banding of chromosome 19[J]. Clin Genet.

[30] Jacobs P, Dalton P, James R, Mosse K, Power M, Robinson D (1997). Turner syndrome: a cytogenetic and molecular study [J]. Ann Hum Genet.

[31] Chauhan P, Jaiswal SK, Lakhotia AR, Rai AK (2016). Molecular cytogenetic characterization of two Turner syndrome patients with mosaic ring X chromosome [J]. J Assist Reprod Genet.

[32] Dutta UR, Vempally S, Ranganath P, Dalal A (2014). A novel combined 15q11.2 duplication and a bisatellited supernumerary marker derived from chromosome 22: molecular characterization of the marker [J]. Gene.

[33] Shaffer LG, Dabell MP, Fisher AJ, Coppinger J, Bandholz AM, Ellison JW (2012). Experience with microarray-based comparative genomic hybridization for prenatal diagnosis in over 5000 pregnancies [J]. Prenat Diagn.

[34] Hay SB, Sahoo T, Travis MK, Hovanes K, Dzidic N, Doherty C (2018). ACOG and SMFM guidelines for prenatal diagnosis: Is karyotyping really sufficient?[J]. Prenat Diagn.

[35] Levy B, Wapner R (2018). Prenatal diagnosis by chromosomal microarray analysis [J]. Fertil Steril.

[36] Desai P, Haber H, Bulafka J, Russell A, Clifton R, Zachary J (2018). Impacts of variants of uncertain significance on parental perceptions of children after prenatal chromosome microarray testing [J]. Prenat Diagn.

[37] Marle N, Martinet D, Aboura A, Joly-Helas G, Andrieux J, Flori E (2014). Molecular characterization of 39 de novo sSMC: contribution to prognosis and genetic counselling, a prospective study [J]. Clin Genet.

[38] Hsu LY, Benn PA, Tannenbaum HL, Perlis TE, Carlson AD (1987). Chromosomal polymorphisms of 1, 9, 16, and Y in 4 major ethnic groups: a large prenatal study [J]. Am J Med Genet.

[39] Clarke AJ, Wallgren-Pettersson C. Ethics in genetic counselling [J]. J Community Genet. 2018. 10.1007/s12687-018-0371-7 PMC6325035 29949066

